# Effect of Fasted Live-Weight Gain during the Cashmere Non-Growing Period on Cashmere Production Performance and Secondary Hair Follicle Activity of Cashmere Goats

**DOI:** 10.3390/ani13223519

**Published:** 2023-11-14

**Authors:** Junxia Li, Wenhui Xing, Tana Gegen, Chunxiang Zhang, Youshe Ren, Chunhe Yang

**Affiliations:** 1 College of Animal Science, Shanxi Agricultural University, Taigu 030801, China; lijunxia0121@163.com (J.L.); xxwzhh2468@163.com (W.X.); chunxiang.zhang@sxau.edu.cn (C.Z.); 2 Agriculture and Animal Husbandry Bureau of Linxi County, Linxi 025250, China; 15661521580@163.com; 3 Key Laboratory of Farm Animal Genetic Resources Exploration and Breeding of Shanxi Province, Taigu 030801, China

**Keywords:** cashmere goat, cashmere non-growing period, fasted live-weight gain, secondary hair follicle activity, cashmere production performance

## Abstract

**Simple Summary:**

Cashmere fiber is characterized by cyclical and seasonal growth, and the growth cycle is divided into a non-growing period (from late spring to late summer) and a growing period (from early autumn to late winter). This study evaluated the effect of fasted live-weight gain during the cashmere non-growing period on cashmere production performance and secondary hair follicle activity. Results of a Pearson correlation analysis showed that fasted live-weight gain during the cashmere non-growing period had a positive correlation with cashmere yield, cashmere staple length, and population of active secondary hair follicles. Cashmere goats with a fasted live-weight gain of 5.0–10.0 kg had a higher cashmere yield, cashmere staple length, and population of active secondary hair follicles than those with a fasted live-weight gain of 0–5.0 kg. Since fasted live-weight gain reflects the nutritional level to a certain extent, this study suggests that nutritional manipulations such as supplementary feeding during cashmere non-growing periods can increase cashmere production performance.

**Abstract:**

The objective of this study was to investigate the effects of fasted live-weight gain during the cashmere non-growing period on cashmere production performance and secondary hair follicle activity, to provide a theoretical basis for appropriate supplementary feeding of cashmere goats. Fifty Inner Mongolian cashmere goats aged 2–4 years old were randomly selected and weighed in May and September 2019, respectively. Based on fasted live-weight gain between the two weights, the experimental ewe goats were divided into two groups: 0–5.0 kg group (*n* = 30) and 5.0–10.0 kg group (*n* = 20). Skin samples and cashmere samples were collected. Results of a Pearson correlation analysis showed that fasted live-weight gain during the cashmere non-growing period had a moderate and strong positive correlation with cashmere yield (*p* = 0.021) and cashmere staple length (*p* = 0.002), respectively, but did not correlate with cashmere diameter (*p* = 0.254). Compared with cashmere goats with a fasted live-weight gain of 0–5.0 kg, cashmere goats with a fasted live-weight gain of 5.0–10.0 kg had a 17.10% increase in cashmere yield (*p* = 0.037) and an 8.09% increase in cashmere staple length (*p* = 0.045), but had no significant difference in cashmere diameter (*p* = 0.324). Results of a Pearson correlation analysis showed that there was a strong positive correlation between fasted live-weight gain and the population of active secondary hair follicles in the skin of cashmere goats (*p* < 0.01). Compared with cashmere goats with a fasted live-weight gain of 0–5.0 kg, cashmere goats with a fasted live-weight gain of 5.0–10.0 kg had an increase in the population of active secondary hair follicles (*p* < 0.05). In conclusion, the fasted live-weight gain during the cashmere non-growing period had a significant effect on secondary hair follicle activity and cashmere production performance in cashmere goats. Since fasted live-weight gain reflects nutritional level to a certain extent, this study suggests that nutritional manipulations such as supplementary feeding during cashmere non-growing periods can increase cashmere production performance. However, specific nutritional manipulations during the cashmere non-growing period need further research to increase cashmere production performance.

## 1. Introduction

Cashmere fiber is an important product of cashmere goats, which is produced by secondary hair follicles in the skin. Cashmere fiber is characterized by cyclical and seasonal growth, which begins to appear and grow on the body surface in summer, continues to grow in autumn to early winter, stops growing in winter, and naturally sheds from the body surface in spring [1]. Therefore, the growth cycle of cashmere fiber is divided into a non-growing period (from late spring to late summer) and a growing period (from late summer to winter) [2,3]. Cashmere yield is determined by length, diameter, and number of cashmere fibers [4,5]. Therefore, an important way to increase cashmere production performance is by increasing cashmere length and number, i.e., promoting the initiation of cashmere growth in the cashmere non-growing period and accelerating the growth in the cashmere growing period [6], and increasing the population of active secondary hair follicles [7].

Numerous studies have shown that cashmere production performance can be significantly improved by appropriately increasing the nutritional level during the cashmere growing period. Increasing the dietary protein level of Liaoning cashmere goats during the cashmere growing period significantly increased cashmere staple length and yield; however, the addition of protein did not increase cashmere staple length when the protein level was increased to a certain level [8]. Feeding Liaoning cashmere goats with high nutrient levels throughout the year significantly increased cashmere yield [9]. The cashmere staple length was significantly higher in all the year-round housed cashmere goats than in grazed cashmere goats [10]. Results of previous studies suggested that appropriately increasing diet and nutritional levels during the cashmere growing period could significantly improve cashmere production performance; however, further increasing nutritional levels could not change cashmere production performance when nutritional levels increased to meet the growth demand of cashmere fiber. Currently, previous studies focused on the effect of nutritional level during the cashmere growing period on cashmere production performance; however, little attention was paid to the effect of nutrition level during the cashmere non-growing period on cashmere production performance. It was reported that, under natural light conditions from May to October, there were no significant differences in cashmere yield, staple length, diameter, and secondary hair follicle activity for cashmere goats with fasted live-weight gain of 8–10 kg [11]. Because fasted live-weight gain reflects the nutrition level to a certain extent [12,13], it is feasible to investigate the effects of nutritional level on cashmere production performance and metabolic activity of secondary hair follicles in cashmere goats by determining fasted live-weight gain during the cashmere non-growing period. 

It is generally believed that secondary hair follicles in the skin of cashmere goats are in the anagen phase from March to September, in the catagen phase from September to December, and in the telogen phase from December to March [14]. Although cashmere fiber does not grow during the cashmere non-growing period, secondary hair follicles in the skin are in the transition stage from telogen to anagen and in the process of structure reconstruction, which determines the numbers of cashmere fibers. Therefore, we hypothesize that the high nutrient level during the cashmere non-growing period may increase secondary hair follicle activity and cashmere production performance. In the present study, we calculated the fasted live-weight gain of cashmere goats during the cashmere non-growing period, then investigated the correlation between fasted live-weight gain and cashmere production performance and secondary hair follicle activity, and further studied the effects of fasted live-weight gain on cashmere production performance and secondary hair follicle activity. This study will provide a theoretical basis for the practice of supplementary feeding of cashmere goats during cashmere non-growing periods to increase cashmere production performance.

## 2. Materials and Methods

In the present study, all experimental procedures were approved by the Institutional Animal Care and Use Committee of Shanxi Agricultural University (Taigu, China, Permission number, SXAU-EAW-2021G. AH.0602001). The experiment was carried out at the YiWei White Cashmere Goat Farm located in the Inner Mongolia Autonomous Region, China (39°06′ N, 107°59′ E).

### 2.1. Animals, Experimental Design, and Management

A total of 50 adult ewe goats with single kids were randomly selected from a flock of 230 Inner Mongolian cashmere goats, including 20 two-year-old goats, 15 three-year-old goats, and 15 four-year-old goats. The experimental ewe goats were weighed in May and September 2019, respectively, and were divided into two groups ranging from 0 to 5.0 kg (*n* = 30) and 5.0 to 10.0 kg (*n* = 20), according to the fasted live-weight gain between the two weights. For each weighing, the animals were fasted overnight after feeding at 1900 hours and were weighed before morning feeding at 0700 hours. The animals were grazed in the pasture from 0800 to 1700 hours and fed in an open barn from 1800 to 0700 hours all year round. The animals were mated in October, kidded in March, and weaned in July. To meet the nutrient requirements of animals during gestation and lactation, the animals were provided with additional supplementary concentrates from January to June. The amount of supplementary feeding was 0.275 kg/day per goat in January, which was gradually increased to 0.4 kg/day in April and subsequently increased to 0.55 kg/day in May and June. The supplementary feed was purchased from Baotou Jiuzhoudadi Biotech Company (Baotou, China) and consisted of 70% corn and 30% concentrate. The animals were combed on 30 April 2020.

### 2.2. Sample Collection

Skin biopsies were excised from the right mid-side flank region in January 2020 using a trephine (1 cm diameter) and were placed on tissue processing cassettes. After 24 h of fixation in 4% neutral formalin solution, the skin samples were dehydrated in ethanol solutions with different concentration gradients, and finally embedded in paraffin for transverse sectioning. Before combing in April 2020, fleece samples within an area of 10 cm × 10 cm were shorn from the left mid-side flank region of each goat for the measurement of fiber staple length and diameter. The total cashmere weight of each goat was recorded after combing in April 2020. 

### 2.3. Cashmere Fiber Measurements

Before washing, cashmere fibers were manually separated from fleece samples containing a mixture of coarse hairs and cashmere fibers and then washed with carbon tetrachloride and distilled water, and finally air dried in a draught cupboard. Cashmere fiber staple length was measured using the ruler method, as described by Yang et al. [15]. For each sample, the staple length of 100 fibers was measured. Cashmere fiber diameter was measured using an optical microscopic projection method, as described by Peterson and Gherardi (1996) [16]. For each sample, the diameter of 200 fibers was measured using an Optic Fiber Diameter Analyzer (CU-6, Beijing United Vision Technical Company, Beijing, China). 

### 2.4. Determination of Indicators Related to Hair Follicle Population

**Figure 1:**
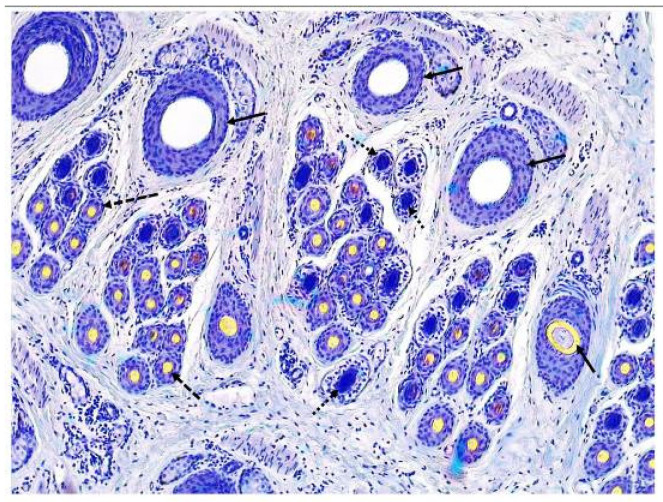
The typical structure of hair follicles in the skin of cashmere goats (10×). **→** This symbol indicates the primary hair follicle; ∙∙∙∙→ this symbol indicates the inactive secondary hair follicle; ---**→** this symbol indicates the active secondary hair follicle.

Skin transverse sections were prepared as described by Yang et al. (2019) [15]. Briefly, transverse sections were serially sectioned at 5 µm thick, parallel to the skin surface, using a rotary microtome (Leica RM2235, Leica, Wetzlar, Germany). Meanwhile, the hair follicle structure was continuously observed. Transverse sections were collected until 5–10 primary follicles with a sebaceous gland appeared. Skin transverse sections were stained using a modified Sacpic method (Nixon, 1993) [17]. Images of hair follicle sections were taken using a microscope camera (Leica ICC 50 W, Leica, Wetzlar, Germany). Ten microscopic fields were captured for each sample, and hair follicles in 10 fields were counted. For each counting, hair follicles that were cut at the top and left margins of the counting area were counted; any that were cut at the bottom and right margins were not included. The primary and secondary hair follicles are easily distinguished, based on their size, arrangement position and accessory structure. Briefly, the primary hair follicles are larger than the secondary hair follicles. In a follicle group, the primary hair follicles are distributed approximately in a straight line on one side and the secondary hair follicles are distributed around primary hair follicles on the other side. The primary hair follicles are accompanied by the sebaceous gland and arrector pili muscle. Active and inactive hair follicles were counted separately, using the method as described by Nixon [17]. Briefly, follicles which contained a fiber, or a fiber canal were considered active; follicles with no fiber canal or which were filled with root sheath cells were considered inactive. Besides the ratio of secondary follicles to primary follicles (S:P) [18], the hair follicle density index (DI) [19], and the hair follicle number (FN) [20] were calculated for indicators of follicle population. Therefore, indicators related to the primary hair follicle population include the primary hair follicle density index (PFDI) and the primary hair follicle number (PFN). Indicators related to the secondary hair follicle population include the secondary hair follicle density index (SFDI), secondary hair follicle number (SFN), and the ratio of secondary follicles to primary follicles (S:P). Since the activity of primary hair follicles is not synchronized and the research focus is not on primary hair follicles and coarse hairs, indicators of primary hair follicle activity are not calculated in the present study. Indicators related to the active secondary hair follicle population include active secondary hair follicle density index (ASFDI), active secondary hair follicle number (ASFN), the ratio of active secondary follicles to primary follicles (Sf:P), and the percentage of active secondary follicles (PASF). The typical structure of hair follicles in the skin of cashmere goats is shown in Figure 1.

### 2.5. Statistical Analysis

All statistical analyses were performed on SAS 9.2 software (SAS Institute Inc., Cary, NC, USA). Pearson correlation analysis between fasted live-weight gain and cashmere production performance (cashmere yield, cashmere fiber staple length, cashmere fiber diameter) and indicators related to active secondary hair follicle population (ASFDI, ASFN, Sf:P, PASF) were performed using the CORR procedure. The correlation is defined as follows: the correlation coefficient |r| ≥ 0.5 is strongly correlated, 0.3 ≤ |r| < 0.5 is moderately correlated, 0.1 ≤ |r| < 0.3 is weakly correlated, and |r| < 0.1 is not correlated [21]. Difference in cashmere production performance (cashmere yield, cashmere fiber staple length, cashmere fiber diameter), indicators related to primary hair follicle population (PFDI, PFN), indicators related to secondary hair follicle population (SFDI, SFN, S:P), and indicators related to active secondary hair follicle population (ASFDI, ASFN, Sf:P, PASF) were estimated using the two independent sample *t*-test procedures. The data are expressed as mean and standard deviation (mean ± SD) and were considered statistically significant and highly significant at *p* < 0.05 and *p* < 0.01, respectively.

## 3. Results

### 3.1. Fasted Live-weight Gain during the Cashmere Non-Growing Period Increased Cashmere Production Performance

To investigate the effects of body weight gain during the cashmere non-growing period on cashmere production performance, we first studied the correlation between body weight gain and cashmere production performance. Results of the Pearson correlation analysis showed that fasted live-weight gain during the cashmere non-growing period had a moderate and strong positive correlation with cashmere yield (r = 0.43, *p* = 0.021) and cashmere staple length (r = 0.55, *p* = 0.002), respectively. The fasted live-weight gain during the cashmere non-growing period did not correlate with cashmere diameter (r = 0.22, *p* = 0.254). Next, differences in cashmere production performance between cashmere goats with fasted live-weight gains of 0–5.0 kg and 5.0–10.0 kg were measured. Compared with cashmere goats with a fasted live-weight gain of 0–5.0 kg, cashmere goats with a fasted live-weight gain of 5.0–10.0 kg had a 17.10% and 8.09% increment in cashmere yield (*p* = 0.037) and cashmere staple length (*p* = 0.045), respectively (Table 1). There was no significant difference in cashmere diameter between the two groups, and the average cashmere diameter of goats in this experiment was (15.03 ± 0.94) μm (Table 1).

### 3.2. Fasted Live-weight Gain during the Cashmere Non-Growing Period Increased the Population of Active Secondary Hair Follicles in the Skin of Cashmere Goats

**Figure 2:**
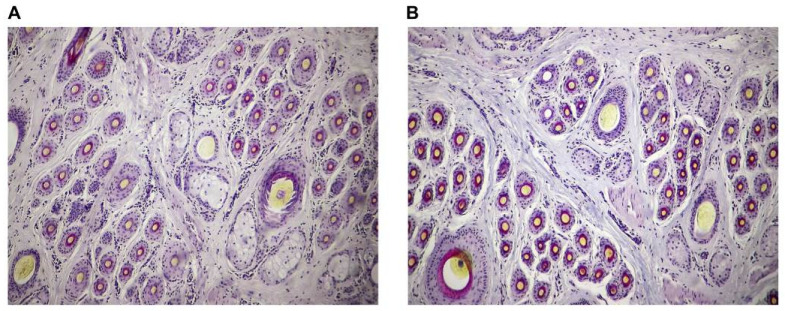
The typical structure of hair follicles in the skin of cashmere goats with a fasted live-weight gain of 0–5.0 kg (**A**) and 5.0–10.0 kg (**B**).

To determine the accuracy of tissue-section and hair-follicle counts, we first studied the effects of fasted live-weight gain during the cashmere non-growing period on populations of primary and secondary hair follicles. There were no differences in indicators related to primary hair follicles (PFDI, PFN) (*p* > 0.05) and secondary hair follicles (SFDI, SFN, and S:P) for cashmere goats with fasted live-weight gains of 0–5.0 kg and 5.0–10.0 kg (*p* > 0.05) (Table 2; Figure 2). In the present study, the PFDI in the skin of cashmere goats was 30.75 ± 4.01, the PFN was (2.94 ± 0.39) million, the SFDI was 441.72 ± 53.93, the SFN was (42.19 ± 5.16) million, and the S:P was 14.91 ± 2.06. The results demonstrated the accuracy of tissue-section and hair-follicle counts. 

The results showed that the fasted live-weight gain during the cashmere non-growing period was strongly positively correlated with active secondary hair follicle populations reflected in ASFN (r = 0.61, *p* = 0.028), ASFDI (r = 0.63, *p* = 0.022), and Sf:P (r = 0.62, *p* = 0.043). Differences in indicators of active secondary hair follicle populations between cashmere goats with a fasted live-weight gain of 0–5.0 kg and 5.0–10.0 kg were estimated. Compared with cashmere goats with a fasted live-weight gain of 0–5.0 kg, cashmere goats with a fasted live-weight gain of 5.0–10.0 kg had an 11.70% increment in ASFN (*p* = 0.039), a 9.80% increment in ASFDI (*p* = 0.059), a 7.27% increment in PASF (*p* = 0.036), and a 24.86% increment in Sf:P (*p* = 0.031) (Table 3; Figure 2).

## 4. Discussion

Cashmere fiber is characterized by cyclical and seasonal growth, and does not grow during the period from the fall of the cashmere (late April and early May) to the regrowth of the body surface (late July and early August). Therefore, the growth cycle of cashmere fiber is divided into a non-growing period (from late spring to late summer) and a growing period (from early autumn to late winter) [2,3]. At present, it has been confirmed that nutrient levels during the cashmere growing period had a significant effect on cashmere production performance; however, the effect of nutrient levels during the cashmere non-growing period on cashmere production performance remains unknown. Because fasted live-weight gain reflects high and low levels of nutrition to a certain extent [12,13], we studied the effects of fasted live-weight gain during the cashmere non-growing period on cashmere production performance and secondary hair follicle activity. The results showed that the fasted live-weight gain during the cashmere non-growing period had a positive correlation with cashmere production performance and secondary hair follicle activity. Furthermore, cashmere goats with a fasted live-weight gain of 5.0–10.0 kg during the cashmere non-growing period had a higher cashmere production performance and secondary hair follicle activity than those with a fasted live-weight gain of 0–5.0 kg.

Previous studies showed that dietary coated methionine supplementation during the cashmere growing period significantly improved cashmere staple length and cashmere yield of Inner Mongolian Cashmere goats [22] and Shanbei White Cashmere goats [23]. To a certain extent, increasing the dietary nutrition level increased the cashmere staple length and cashmere yield; however, when the nutrient level was increased to exceed the need for cashmere growth, increasing the nutrient level did not affect cashmere production performance [8]. It was reported that the cashmere staple length of cashmere goats which were house-fed all year round was 0.36 cm longer than that of grazing cashmere goats [10]. Feeding diets with high nutrient levels throughout the year significantly increased the body weight, cashmere yield, and cashmere staple length of Liaoning cashmere goats [9]. Based on a comprehensive analysis of the above research results, supplementary feeding during the cashmere non-growing period may contribute to the increase in cashmere staple length and the cashmere yield of cashmere goats given high nutrient-level diets throughout the year. The results of this present experiment preliminarily support the above inference. In the present study, the cashmere yield and staple length of cashmere goats with a fasted live-weight gain of 5.0–10.0 kg were significantly higher than those of cashmere goats with a fasted live-weight gain of 0–5.0 kg during the cashmere non-growing period. Cashmere yield is determined by cashmere staple length, cashmere diameter, and cashmere number [24]. In the present study, the cashmere yield and staple length of cashmere goats with a fasted live-weight gain of 5.0–10.0 kg were 17.10% and 8.09% higher than those of cashmere goats with a fasted live-weight gain of 0–5.0 kg, respectively. However, there was no difference in cashmere diameter between the two groups. Since the increase in cashmere yield is not proportional to the increase in cashmere staple length, it suggests an increase in cashmere number. During the cashmere growing period, the number of cashmere fibers is equal to the population of active secondary hair follicles. Therefore, it is necessary to study the effect of body weight gain during the cashmere non-growing period on the population of active secondary hair follicles in the skin of cashmere goats.

It has been confirmed that the morphogenesis of primary hair follicles and secondary hair follicles in the skin of cashmere goats begins from the fetal stage; primary hair follicles are fully developed at birth, and their populations do not change thereafter [25]. Only a part of the secondary hair follicle matures at birth and produces cashmere fibers; most of them mature at 3–6 months after birth, and populations of secondary hair follicles do not change thereafter [26]. Previous studies showed that feeding high levels of crude protein during the cashmere growing period had no significant effect on populations of primary hair follicles in cashmere goats [27]. Similar results were obtained in the present study. In the present study, there was no significant difference in populations of primary hair follicles in the skin of cashmere goats with fasted live-weight gains of 0–5.0 kg and 5.0–10.0 kg, which was consistent with the results of Denny et al. [28]. The results of this study and previous studies showed that populations of primary hair follicles in the skin of cashmere goats were not affected by dietary nutrition levels. In the present study, there was no significant difference in populations of secondary hair follicles in the skin of cashmere goats with fasted live-weight gains of 0–5.0 kg and 5.0–10.0 kg, indicating that nutritional level did not affect populations of secondary hair follicles in adult cashmere goats, which was consistent with the results of Zhao et al. [27]. It was reported that feeding high protein level diets to Longdong cashmere goats during the cashmere growing period did not change populations of secondary hair follicles [27]. The results of the present study and previous studies showed that, for adult cashmere goats, the dietary nutrient level during the cashmere growing and non-growing period did not change the populations of secondary hair follicles in the skin of cashmere goats, indicating that populations of secondary hair follicles in the skin of cashmere goats do not change throughout their life once they are mature. Meanwhile, the results of this study showed that the hair-follicle sectioning technique in the present study was reliable, and provided a reliable condition for counting populations of active secondary hair follicles in the subsequent analysis.

The morphogenesis of secondary hair follicles in the skin of cashmere goats initiates in the fetal period; only a part of the secondary hair follicles is mature at birth, and most secondary hair follicles mature at the age of 3–6 months after birth [29]. Once secondary hair follicles are mature, the populations do not change; they go through a complete growth period, and then enter a cyclical change process, that is, they continuously undergo catagen, telogen, and anagen phases [30]. Due to the influence of breed, age, sex, nutritional level, experimental operation, and other factors, the periodic change time of secondary hair follicles in the skin of cashmere goats is slightly different. It is generally believed that the secondary hair follicles in the skin of cashmere goats are in the anagen phase from March to September, in the catagen phase from September to December, and in the telogen phase from December to March [14]. Previous studies showed that fasted live-weight gain of cashmere goats fed high-energy-level and low-energy-level diets ranged from 8 to 10 kg under natural light conditions from May to October, and there was no significant difference in secondary hair follicle activity between the two groups [11]. Similar results were obtained in the present study. In this study, there was a strong positive correlation between fasted live-weight gain during the cashmere non-growing period and populations of active secondary hair follicles in the skin of cashmere goats. The populations of active secondary hair follicles in the skin of cashmere goats with a fasted live-weight gain of 5.0–10.0 kg was significantly higher than those in the skin of cashmere goats with a fasted live-weight gain of 0–5.0 kg. The results of this experiment suggested that improving the nutritional level of cashmere goats during the cashmere non-growing period could significantly increase the metabolic activity of secondary hair follicles, increase the populations of active secondary hair follicles, and then improve cashmere production performance. However, combined with the results of this experiment and those of Zhang et al. [11], the metabolic activity of secondary hair follicles could not be further improved when the dietary nutrient level was increased to a certain limit. It was reported that different levels of crude-protein supplementation during the cashmere growing period had no significant effect on populations of active secondary hair follicles in Longdong cashmere goats, indicating that secondary hair follicle activity had been determined after completion of the reconstruction of the secondary hair follicle structure and that then increasing the nutritional level could not change populations of active secondary hair follicles [27]. Although cashmere fiber does not grow during the cashmere non-growing period, secondary hair follicles in the skin are in the transition stage from telogen to anagen and in the process of structure reconstruction [14,31], which determines the populations of cashmere fiber. The results of this study suggested that nutritional manipulations such as supplementary feeding during the cashmere non-growing period could significantly increase the activity of secondary hair follicles and then increase cashmere production performance. However, specific nutritional manipulations during the cashmere non-growing period need further research to increase cashmere production performance.

## 5. Conclusions

The fasted live-weight gain of cashmere goats during the cashmere non-growing period had a positive correlation with cashmere production performance and secondary hair follicle activity. Cashmere goats with a fasted live-weight gain of 5.0–10.0 kg during the cashmere non-growing period had a higher cashmere production performance and secondary hair follicle activity. This study suggests that nutritional manipulations such as supplementary feeding during cashmere non-growing periods can increase cashmere production performance. However, specific nutritional manipulations during the cashmere non-growing period need further research to increase cashmere production performance. 

## Figures and Tables

**Table 1:** Effect of fasted live-weight gain during the cashmere non-growing period on cashmere production performance in cashmere goats.

Group	Cashmere Yield (g)	Cashmere Staple Length (cm)	Cashmere Diameter (μm)
0–5.0 kg	718.0 ± 124.9	8.91 ± 0.83	14.87 ± 0.94
5.0–10.0 kg	840.8 ± 165.4	9.64 ± 1.00	15.22 ± 0.94
*p* value	0.037	0.045	0.324

**Table 2:** Effect of fasted live-weight gain during the cashmere non-growing period on populations of the total secondary and primary hair follicles in cashmere goats.

Group	PFDI	PFN	SFDI	SFN	S:P
0–5.0 kg	31.17 ± 4.48	2.98 ± 0.43	443.00 ± 62.72	42.41 ± 6.02	14.38 ± 2.17
5.0–10.0 kg	30.09 ± 4.03	2.86 ± 0.39	439.10 ± 49.41	41.74 ± 4.66	15.76 ± 2.02
*p* value	0.669	0.630	0.917	0.849	0.277

Note: PFDI, Primary Follicle Density Index; PFN, Primary Follicle Number; SFDI, Secondary Follicle Density Index; SFN, Secondary Follicle Number; S:P, ratio of secondary to primary follicle.

**Table 3:** Effect of fasted live-weight gain during the cashmere non-growing period on populations of active secondary hair follicles in cashmere goats.

Group	ASFDI	ASFN	S*f*:P	PASF
0–5.0 kg	329.70 ± 14.50	31.54 ± 1.52	10.62 ± 1.47	81.01 ± 3.13
5.0–10.0 kg	362.0 ± 32.13	35.23 ± 3.03	13.26 ± 1.71	86.90 ± 1.87
*p* value	0.059	0.039	0.031	0.036

Note: ASFDI, Active Secondary Follicle Density Index; ASFN, Active Secondary Follicle Number; S*f*:P, ratio of active secondary to primary follicle; PASF, Percentage of Active Secondary Follicles.

## Data Availability

Data is contained within the article.
